# Supplementary figures and images for: The Legacy of Hg Contamination in a Past Mining Area (Tuscany, Italy): Hg Speciation and Health Risk Assessment
Source: Toxics. 2024 Jun 16;12(6):436. doi: 10.3390/toxics12060436 (PMC11209415; doi:10.3390/toxics12060436)

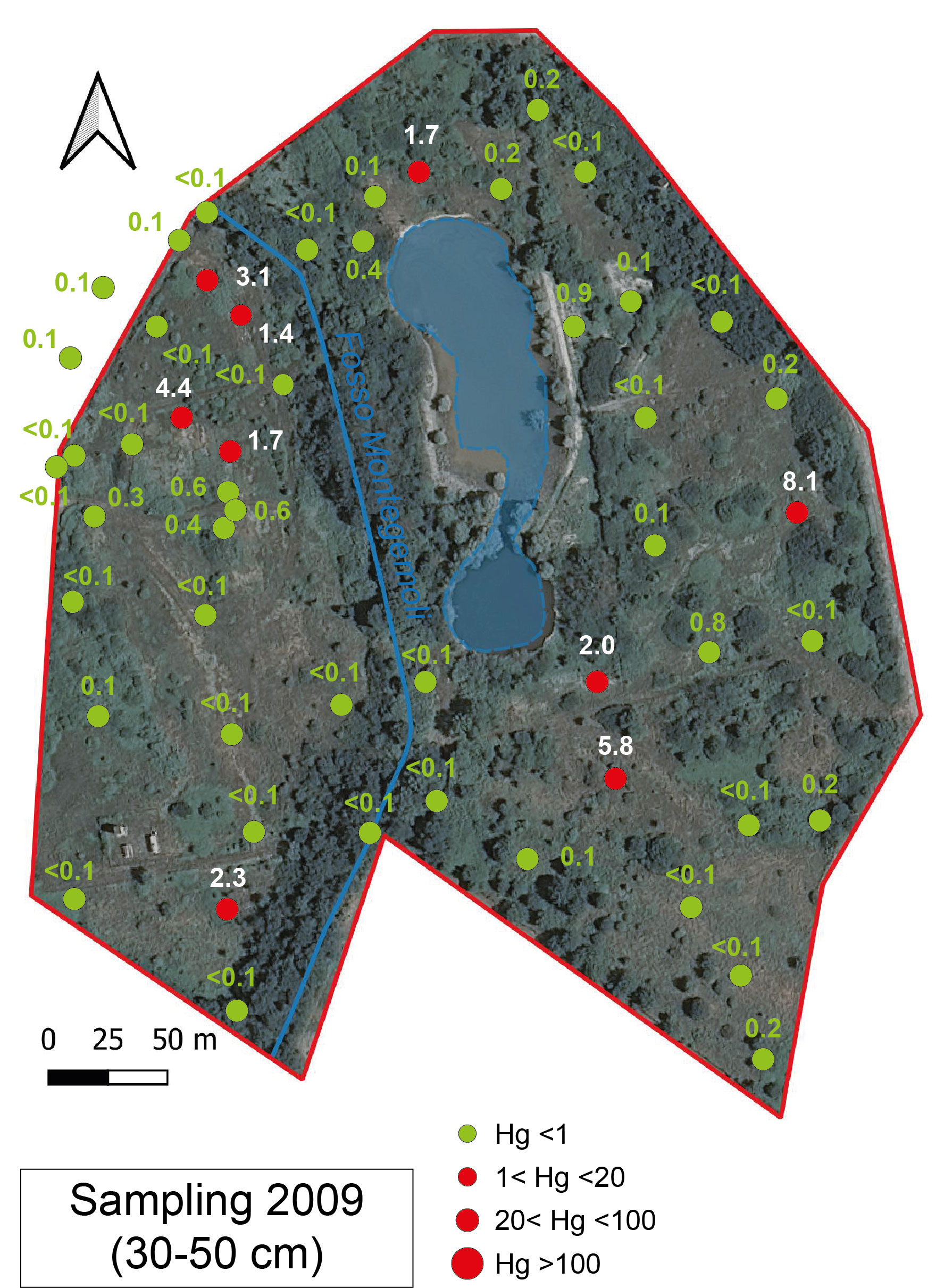

Supplement: Supplementary file 1 [file toxics-12-00436-s001.zip › Figure S1.jpg]
